# Pre- and postsynaptic changes in the neuromuscular junction in dystrophic mice

**DOI:** 10.3389/fphys.2015.00252

**Published:** 2015-09-09

**Authors:** Stephen J. P. Pratt, Ana P. Valencia, Gloribel K. Le, Sameer B. Shah, Richard M. Lovering

**Affiliations:** ^1^Department of Orthopaedics, University of Maryland School of MedicineBaltimore, MD, USA; ^2^Department of Kinesiology, University of Maryland School of Public HealthCollege Park, MD, USA; ^3^Departments of Orthopaedic Surgery and Bioengineering, University of CaliforniaSan Diego, La Jolla, CA, USA

**Keywords:** *mdx*, synaptophysin, subsynaptic nuclei, NMJ occupancy, neurofilament, neuron

## Abstract

Duchenne muscular dystrophy (DMD) is a devastating neuromuscular disease in which weakness, increased susceptibility to muscle injury, and inadequate repair appear to underlie the pathology. While most attention has focused within the muscle fiber, we recently demonstrated in *mdx* mice (murine model for DMD) significant morphologic alterations at the motor endplate of the neuromuscular junction (NMJ) and corresponding NMJ transmission failure after injury. Here we extend these initial observations at the motor endplate to gain insight into the pre- vs. postsynaptic morphology, as well as the subsynaptic nuclei in healthy (WT) vs. *mdx* mice. We quantified the discontinuity and branching of the terminal nerve in adult mice. We report *mdx*- and age-dependent changes for discontinuity and an increase in branching when compared to WT. To examine *mdx*- and age-dependent changes in the relative localization of pre- and postsynaptic structures, we calculated NMJ occupancy, defined as the ratio of the footprint occupied by presynaptic vesicles vs. that of the underlying motor endplate. The normally congruent coupling between presynaptic and postsynaptic morphology was altered in *mdx* mice, independent of age. Finally we found an almost two-fold increase in the number of nuclei and an increase in density (nuclei/area) underlying the NMJ. These outcomes suggest substantial remodeling of the NMJ during dystrophic progression. This remodeling reflects plasticity in both pre- and postsynaptic contributors to NMJ structure, and thus perhaps also NM transmission and muscle function.

## Introduction

The most common and severe form of muscular dystrophy is Duchenne muscular dystrophy (DMD), a disorder caused by the absence of dystrophin, a structural protein found on the cytoplasmic surface of the sarcolemma (Watkins et al., 1988). The *mdx* mouse also lacks dystrophin and has been widely used as an animal model of DMD (Bulfield et al., 1984; Willmann et al., 2009). Dystrophin is part of the dystrophin-associated glycoprotein complex (DGC or DAPC), which connects the internal cytoskeleton of the muscle fiber to the extracellular matrix. The DGC also accumulates at the postsynaptic membrane (aka motor endplate) of the neuromuscular junction (NMJ), the area of synaptic contact between a motor neuron and its target muscle fiber (Grady et al., 2000). The motor endplate is a specialized area of the sarcolemma that rapidly and consistently responds to release of neurotransmitter from the overlying nerve terminal. Proper development and organization at the NMJ are necessary for effective neuromuscular transmission (Wood and Slater, 1997; Sanes and Lichtman, 1999), but a number of pathological conditions affecting the distribution of acetylcholine receptors (AChRs) can lead to impaired transmission (Wood and Slater, 2001). The NMJ can display alterations in synaptic organization due to inactivity, denervation, aging, or crush injury to the nerve/muscle (Wilson and Deschenes, 2005; Jang and Van Remmen, 2011; Kawabuchi et al., 2011; Sieck et al., 2012). Similarly, the absence of associated proteins can cause changes in structure, and without exception, the motor endplate is noticeably disrupted in patients with DMD and *mdx* mice (Kong and Anderson, 1999; Adams et al., 2000; Marques et al., 2004; Banks et al., 2009; Chipman et al., 2010; Kulakowski et al., 2011).

Patients with DMD and *mdx* mice also have increased susceptibility to injury compared to their non-dystrophic counterparts. Over time, this damage/degeneration exceeds the ability to repair/regenerate muscle, leading to irreversible muscle wasting throughout life. The increased force loss after contraction-induced injury is typically attributed to structural weakness of the muscle fiber cytoskeleton and changes in signaling within myofibers secondary to the loss of dystrophin (Hoffman et al., 1987; Lovering et al., 2009). However, we have previously reported that loss in whole muscle function after injury is also associated with alterations in NMJ endplate morphology and synaptic transmission in *mdx* mice (Pratt et al., 2013, 2015). Such structural and functional changes may be another contributor to the greater force loss seen after injury (i.e., muscle fragility) in dystrophic muscle. Based on these findings, we hypothesized that the absence of dystrophin in muscle not only affects the motor endplate, but also the spatial relationship and organization among the terminal axon, presynaptic terminal bouton and postsynaptic membrane. In this study, we further examined changes in NMJ morphology between WT and *mdx* muscle. Our data support a model where both pre- and postsynaptic structural abnormalities in the NMJ of *mdx* mice contribute to deficits in NMJ function and muscle contractility, ultimately impairing neuromuscular health.

## Methods

### Animals

We used age-matched male control (WT) and *mdx* (lacking dystrophin) mice from the C57BL/10ScSnJ strain (The Jackson Laboratory, Bar Harbor, ME). A total of 14 mice were used (3 months of age, *N* = 8 and 6 months of age, *N* = 6). All experimental procedures were approved by the University of Maryland Institutional Animal Care & Use Committee.

### Assessment of subsynaptic nuclei at the motor endplate

Animals were perfusion-fixed through the left ventricle with 4% paraformaldehyde (3 months of age: WT, *N* = 1; *mdx*, *N* = 1). Quadriceps muscles were dissected and stored in fixative for 24–48 h until stained with α-Bungarotoxin (BTX, identifies the acetylcholine receptors of the postsynaptic membrane of the NMJ) conjugated to Alexa Fluor-488 (B13422, Life Technologies, Carlsbad, CA) at a 1:200 dilution in phosphate buffered saline (PBS) for 1 h. The quadriceps whole mount tissue preparations were then mounted in Vectashield antifade mounting medium with DAPI (4′,6-diamidino-2-phenylindole, identifies nuclei) (H-1200, Vector Laboratories, Bulingame, CA) and firmly coverslipped using tape. Digital images of NMJs from whole mount tissue preparations were obtained with a Zeiss 510 confocal laser-scanning microscope (63x objective, zoom 2) with pinhole set at 1.0 Airy unit. Only NMJs in a complete *en face* view were selected for analysis (WT, *N* = 27; *mdx N* = 21). This was confirmed using 3 dimensional rotating images of NMJs, reconstructed from confocal Z-stacks (via the projection application in the Zeiss LSM image browser software). To encompass NMJs at different depths in the whole mount, laser intensity and scan settings, for each color channel, were adjusted on a sample-to-sample basis until signal saturation was achieved, as indicated using the range indicator in LSM software. Confocal Z-stacks of the entire postsynaptic membrane at the NMJ were acquired, and only nuclei present within this range were identified as subsynaptic (Simon et al., 1992; Briguet and Ruegg, 2000) and included for analysis. A maximum intensity flat plane projection was then made from Z-stacked images in Image J software (NIH) to account for the depth of the NMJ in measurements. After background was subtracted and noise despeckled, a Gaussian Blur filter with σ = 2.00 was applied. Binary images were then generated. For each image, the motor endplate was outlined using freehand tools in Image J and identified as the region of interest (ROI) which encompasses the acetylcholine receptor clusters. Nuclei that were completely within, as well as those partially crossing, the boundary of the ROI were quantified for each NMJ. To account for differences in the number of nuclei among NMJs, and between groups, as a result of size differences in the motor endplate, the number of nuclei quantified was normalized to total area of the endplate, for each NMJ.

### Nerve morphology and presynaptic to postsynaptic occupancy

**Figure 1:**
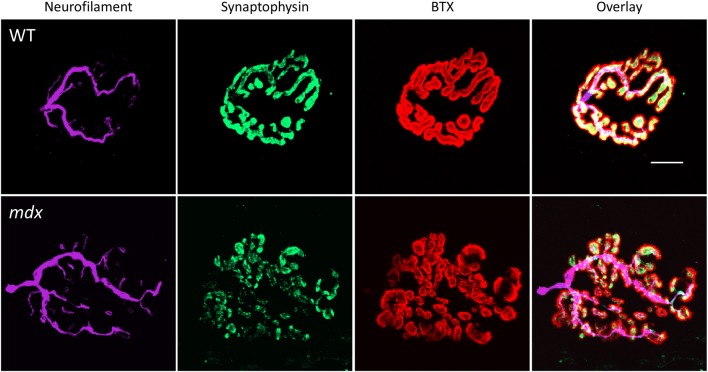
**Representative images of healthy and dystrophic neuromuscular junctions**. Images show confocal Z-stacks of neuromuscular junctions (NMJ) from both a wild type (WT) and dystrophic (*mdx*) mouse. Antibodies against neurofilament (terminal nerve, purple) and synaptophysin (presynaptic vesicles, green) and α-bungarotoxin staining (BTX, postsynaptic acetylcholine receptors, red) were used to image the complete structure of the NMJ. The boundaries of the Z-stack were defined by the upper limit of neurofilament labeling and the lower limit of BTX staining. Scale bar = 10 μm.

Whole mount tissue preparations from the quadriceps were acquired as above (3 months of age: WT, *N* = 3; *mdx*, *N* = 3. 6 months of age: WT, *N* = 4; *mdx*, *N* = 2). Samples were then permeabilized with 0.5% triton in PBS for 30 min and blocked with 3% bovine serum albumin (BSA) in PBS for 1 h, both at room temperature. Samples were incubated simultaneously with primary antibodies against synaptophysin (mouse monoclonal, M7315, Dako, Carpinteria, CA) at a 1:50 dilution in PBS and neurofilament heavy chain (chicken polyclonal, CPCA-NF-H, EnCor Biotechnology Inc., Gainesville, FL) at a 1:1000 dilution in PBS overnight at 4°C. After washing 3 times for 10 min each, goat anti-mouse (conjugated to Alexa Fluor-488, A21235, Life Technologies, Carlsbad, CA), and goat anti-chicken (conjugated to Alexa Fluor-647, A21449, Life Technologies, Carlsbad, CA) secondary antibodies were diluted 1:100 in PBS and applied to samples for 2 h at 4°C. At the time of incubation with secondary antibodies, samples were also incubated with BTX conjugated to Alexa Fluor-555 (B35451, Life Technologies, Carlsbad, CA) at a 1:200 dilution. Finally, samples were washed 3 times at 10 min each, mounted in Vectashield antifade mounting medium (H-1000, Vector Laboratories, Burlingame, CA), and firmly coverslipped using tape. Confocal Z-stacks were acquired as above, however the limits of the Z-stack were identified using the upper limit of neurofilament labeling (NF-H) (Figure 1, purple) and the lower limit of postsynaptic staining (BTX) (Figure 1, red). Presynaptic labeling (synaptophysin) (Figure 1, green) present within this range was also used for analysis. Images were then processed in Image J as described above, from which pixel area (μm^2^) was measured. NMJ occupancy (Dachs et al., 2011; Sleigh et al., 2014) was calculated as a percentage of presynaptic area vs. postsynaptic area (pre/post * 100) (NMJs from 3 month old mice: WT, *N* = 28; *mdx N* = 26. NMJs from 6 month old mice: WT, *N* = 34; *mdx N* = 24). Images of neurofilament were further processed as described (Pratt et al., 2013). Briefly, binary images were skeletonized and a histogram describing the connectivity for each pixel were generated using the BinaryConnectivity.class Image J plugin (http://www.dentistry.bham.ac.uk/landinig/software/software.html). Histogram bins correspond to the number of neighboring pixels for each pixel. One neighbor implies a terminal pixel, two neighbors imply a pixel along a single branch, and three or more neighbors indicate that a pixel exists at a branch node. Thus, discontinuities (terminal pixel) or branching (3+ neighbors) were quantified (*Discontinuity*. NMJs from 3 month old mice: WT, *N* = 17; *mdx N* = 24. NMJs from 6 month old mice: WT, *N* = 30; *mdx N* = 18) (*Branching*. NMJs from 3 month old mice: WT, *N* = 17; *mdx N* = 24. NMJs from 6 month old mice: WT, *N* = 23; *mdx N* = 15).

### Statistical analysis

To evaluate differences between groups a Two-Way ANOVA was used (SigmaStat, San Rafael, CA). A Holm-Sidak post hoc analysis was performed to determine where significant differences had occurred. Significance was set at *p* < 0.05.

## Results

**Figure 2:**
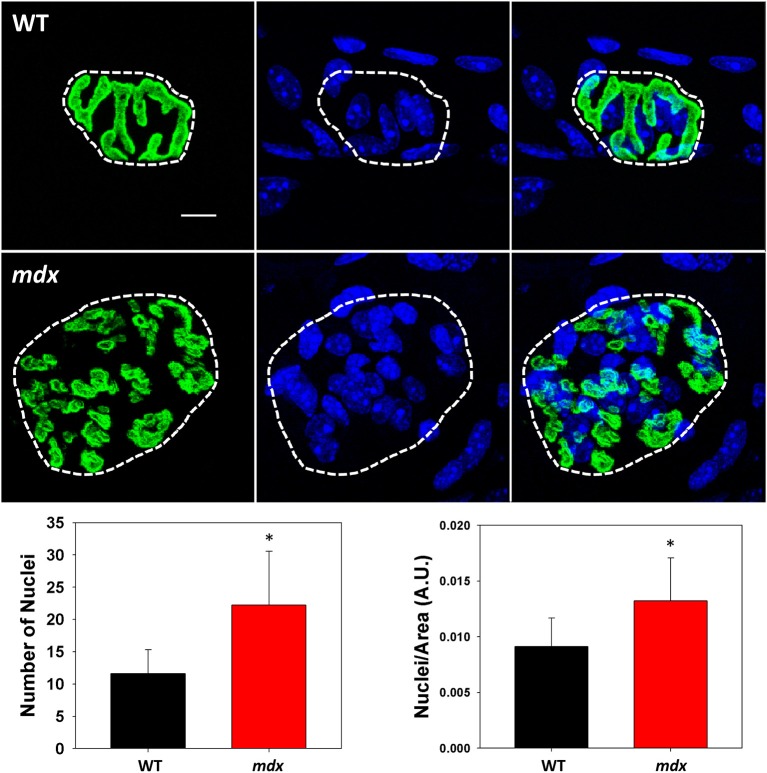
**NMJ and nuclei**. Images of wild type (WT) and dystrophic (*mdx*) NMJs from quadriceps whole mount preparations showing the postsynaptic structure (green, BTX) and the subsynaptic nuclei (blue, DAPI). Corresponding bar graphs show quantification of nuclei specifically at the motor endplate (dotted line) as well as counts of nuclei normalized to endplate area. Data collected from 48 NMJs (27 WT, 21 *mdx*, 3 months of age). ^*^Indicates significance compared to wild type (*p* < 0.05). Scale bar = 10 μm.

Motor endplate fragmentation is seen independent of injury in a variety of hindlimb and forelimb muscles of dystrophic mice (Lyons and Slater, 1991; Kong and Anderson, 1999; Santo Neto et al., 2003; Banks et al., 2009). Dystrophin is not required for NMJ formation, but is thought to be required for endplate maintenance (Kong and Anderson, 1999), as the motor endplate is significantly altered in *mdx* muscle compared to the NMJ in WT muscle (Figure 2, green). Interestingly, the nuclei underlying NMJs from dystrophic muscle (Figure 2, blue) are increased in number (from 12 ± 4 to 22 ± 8, WT vs. *mdx*, respectively) and density (from 0.009 ± 0.003 to 0.013 ± 0.004, WT vs. *mdx* respectively) (Figure 2, bar graphs).

**Figure 3:**
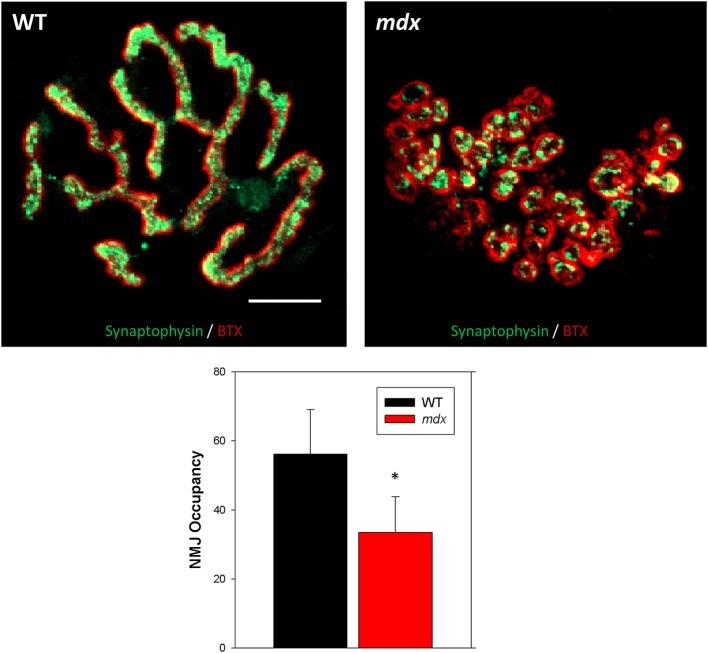
**NMJ occupancy**. Overlay images of wild type (WT) and dystrophic (*mdx*) NMJs showing both the presynaptic terminal bouton (green, synaptophysin) and postsynaptic acetylcholine receptors (red, BTX). NMJ occupancy was calculated as, (presynaptic area/postsynaptic area)^*^100. Data collected from 58 NMJs (34 WT, 24 *mdx*). ^*^Indicates significance compared to wild type (*p* < 0.05). Scale bar = 10 μm.

Structure is clearly a major determinant of function in biology, especially in muscle. In the same way that the development of force relies on the controlled overlap of actin and myosin, the apposition of the nerve terminal and the underlying motor endplate is likely a major determinant of NMJ function. NMJ occupancy describes the overlap between the presynaptic and postsynaptic morphology of the NMJ, which in healthy muscle is high. We used antibodies against synaptic vesicles (synaptophysin) to image the terminal bouton of the neuron and BTX to stain acetylcholine receptors of the motor endplate (Figure 3, green and red, respectively). The orientation of these structures is altered in *mdx* muscle such that the ratio (percent occupancy) of pre-to postsynaptic area is significantly reduced (WT, 56.2 ± 12.9 %; *mdx*, 33.5 ± 10.3%). Despite this reduction in pre- to postsynaptic ratio, the overall alignment of the presynaptic terminal bouton and postsynaptic motor endplate remains.

**Figure 4:**
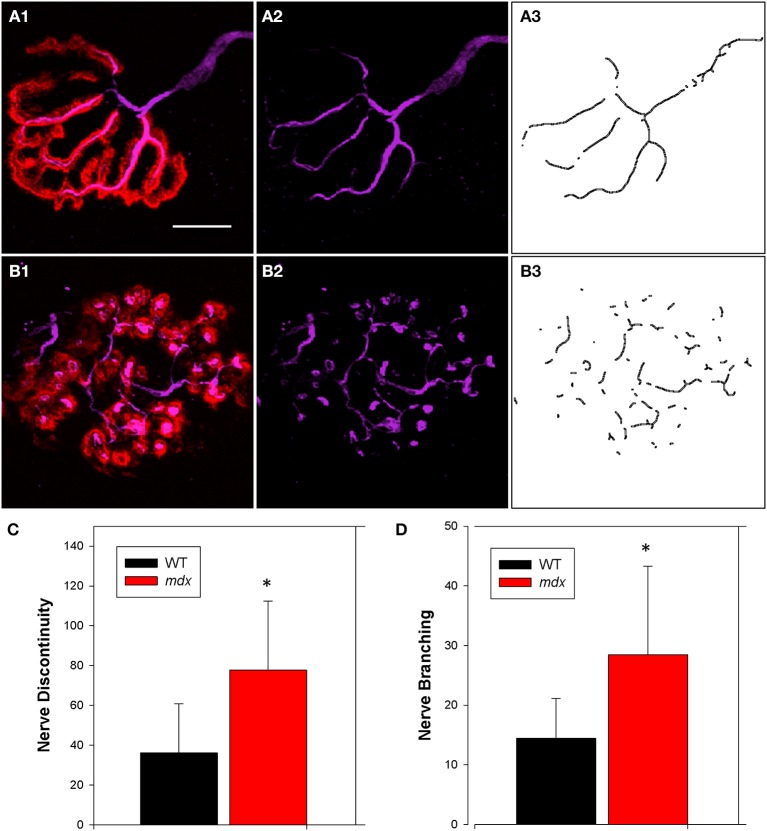
**Neuronal morphology. (A1,B1)** Overlay images of wild type (WT, **A1–A3**) and dystrophic (*mdx*, **B1–B3**) NMJs showing the terminal aspect of the neuron (purple, neurofilament) and postsynaptic acetylcholine receptors (red, BTX). **(A2,B2)** Shows examples of the neuron before processing, followed by skeletonization of digital images post-processing **(A3,B3)**. Binary images were skeletonized and the corresponding histograms describing the discontinuity **(C)** and branching **(D)** were generated as described in the Methods. Data collected from 48 NMJs (30 WT, 18 *mdx*). ^*^Indicates significance compared to wild type (*p* < 0.05). Scale bar equals 10 μm.

We also examined the terminal aspect of the neuron (using antibodies against neurofilament, Figure 4, purple) in close proximity to the motor endplate (using BTX, Figure 4, red). Composite images of confocal Z-stacks were processed and analyzed using Image J. We found intra-terminal and extra-terminal (aka pre-terminal) branching (axonal sprouting) as reported by others (Santo Neto et al., 2003; Marques et al., 2007) (Figure 4B, purple). However, quantification in the current study provides significant data showing a lack of continuity (Figure 4C) and an increase in branching (Figure 4D). All morphological data are summarized in Table 1.

**Table 1:** **Summary of NMJ morphological characteristics**.

**Age**	**WT**		***mdx***	
	**3 months**	**6 months**	**3 months**	**6 months**
SV μm^2^	209.3±77.5	216.4±65.4	147.4±75.8^*^	189.4±90.6^*^
AChRs μm^2^	340.7±122.9	394.0±132.0^†^	458.8±198.8^*^	577.0±277.3^*^^†^
Nerve μm^2^	120.1±50.8	129.8±59.3	134.8±67.1	173.1±127.8
NMJ occupancy (%)	59.4±17.2	56.2±12.9	35.3±16.8^*^	33.5±10.3^*^
Discontinuity	15.6±6.8	36.1±24.7^†^	38.4±24.6^*^	77.7±34.6^*^^†^
Branching	11.7±7.7	14.3±6.7	21.5±14.1^*^	28.4±14.8^*^

Data were derived from wild type (WT) and dystrophic (mdx) mouse quadriceps muscles at 3 months and 6 months of age. Confocal Z-stack images of NMJs were processed (as described in Methods) and area measured for the terminal nerve, presynaptic structure (synaptic vesicles, SV) and postsynaptic endplate (acetylcholine receptors, AChRs). NMJ occupancy is calculated as (presynaptic area/postsynaptic area) ^*^ 100. Discontinuity and branching at the terminal nerve were derived from skeletonized images of the nerve (as described in Methods).

* *Indicates significance compared to age-matched wild type (p < 0.05)*.

† *Indicates significance between young and adult (p < 0.05)*.

## Discussion

The genetic basis for DMD has been determined (Wagner, 2002; Lovering et al., 2005; McNally and Pytel, 2007), but the mechanisms responsible for the progressive decrease in muscle specific force and increased susceptibility to injury are still being clarified. The increased force loss after injury is hypothesized to be due to structural weakness of the muscle fiber cytoskeleton or changes in signaling secondary to the loss of dystrophin (Lovering et al., 2009).

Bidirectional communication between muscle fibers and motor neurons is extremely important for maintenance of the neuromuscular apparatus (Grinnell, 1995). Effective neurotransmission depends upon the regular arrangement of postsynpatic AChRs and proper alignment between the terminal bouton and underlying motor endplate. The absence of dystrophin causes motor endplate fragmentation as evidenced by staining of AChRs, but dystrophin (Kong and Anderson, 1999) is not required for the formation and clustering of AChRs. In addition to muscle-dependent changes in the motor endplate, neuron-dependent mechanisms are also involved in the changes in acetylcholine receptor distribution in young dystrophic muscles (Marques et al., 2007).

Motor endplate fragmentation is typical in adult dystrophic mice and, although some have suggested that the altered morphology is secondary to the destabilization of the sarcolemma and cytoskeleton in *mdx* muscle (Banks et al., 2009), others suggest that the disrupted morphology is the consequence of myofiber degeneration and regeneration (Minatel et al., 2001; Li et al., 2011). Axonal sprouts are induced in the presence of regenerating muscle fibers (van Mier and Lichtman, 1994) and because cycles of degeneration-regeneration occur in patients with DMD and in *mdx* mice, the incidence of sprouting is expected to be higher in dystrophic motor neurons. While there is an increase in the amount of intra-terminal and extra-terminal sprouting during the normal course of the disease in the *mdx* mice (Lyons and Slater, 1991), no collateral axonal sprouting has been reported in DMD patients (Coërs and Telerman-Toppet, 1977). Santo et al. have suggested that such findings, i.e., a reduced or absent axonal sprouting capacity in muscular dystrophy, could partially underlie the poor functional recovery seen after cell-based therapies (Santo Neto et al., 2003).

Interestingly, our data are also consistent with a more active neuronal role in NMJ fragmentation and remodeling, beyond the passive compensatory response to cyclic muscle degeneration implied in the above studies. Indeed, several studies reveal a strikingly similar fragmentation phenotype in response to denervation in the absence of cyclic muscle degeneration-regeneration, including damage to the nerve cell body (Kuromi and Kidokoro, 1984), peripheral nerve injury (Apel et al., 2009), or age-dependent denervation (Valdez et al., 2010). Further, the delivery of certain truncated dystrophins and resulting rescue of muscle degeneration, does not prevent NMJ fragmentation in *mdx* mice (Banks et al., 2009).

In terms of functional impact, the relative roles of myofiber injury, degeneration, and denervation that contribute to the changes seen in NMJ morphology of dystrophic muscles are not yet known, but will be important to elucidate, as NMJ dysfunction is fundamental to understanding impairment of muscle. Morphological changes in the NMJ, together with changes in NMJ transmission, can result in excitation–contraction impairment, causing a decline in muscle contractility (Pratt et al., 2013). Animal studies have shed light on the importance of NMJ structure, but direct study of NMJ in humans remains extremely challenging. There is great interest in the changes that occur at the NMJ with aging (Deschenes et al., 2011, 2013; Jang and Van Remmen, 2011; Punga and Ruegg, 2012; Gonzalez-Freire et al., 2014; Rudolf et al., 2014) and in aging muscle it remains unclear whether denervation precedes sarcopenia, or vice versa (Gonzalez-Freire et al., 2014). Though our data did not reveal any changes in NMJ morphology between young (3 months) and mature (6 months) mice, save for a slight increase in terminal nerve fragmentation (Table 1), there is strong evidence that changes in endplate morphology and NMJ remodeling occur with aging, and even precede loss of motor units (Gonzalez-Freire et al., 2014). However, it is unlikely that there is a change in NMJ occupancy, as presynaptic to postsynaptic relationships are tightly maintained with aging (Deschenes et al., 2013). On the other hand, synaptic transmission becomes more variable with age in the *mdx* mouse model of DMD (Carlson and Roshek, 2001; Kawabuchi et al., 2011), which could provide one explanation why, despite the consistent lack of dystrophin, *mdx* skeletal muscle generates less specific force and becomes more susceptible to damage with age (Chan et al., 2007). Such dramatic NMJ structural and functional impairment in the *mdx* mouse may reflect contributions from changes in the spatial relationship between pre- and postsynaptic structures.

In the current study, we report a decrease in synaptic vesicle area in *mdx* mice compared to WT. This finding is in agreement with an earlier study in *mdx* mice showing a decrease in presynaptic area (Torres and Duchen, 1987), but differs from other studies showing no change (Lyons and Slater, 1991) in *mdx* mice and in Duchenne patients (Jerusalem et al., 1974). It is possible that the discrepancy lies in the methods, as these studies used electron microscopy of multiple muscle sections for area measurements, while the current study used one image to measure area. Similarly, two of these studies report a decrease in post- to presynaptic length ratios (Jerusalem et al., 1974; Torres and Duchen, 1987), while Lyons and Slater report no differences in occupancy (Lyons and Slater, 1991). Here, we show a reduction in NMJ occupancy that was due to both a reduction in presynaptic area and an increase in postsynaptic area, when compared to WT.

An unexpected finding was that the nuclei underlying NMJs are increased in number and density in dystrophic muscles. Preferential transcription of synaptic genes occurs in these synapse-associated nuclei (i.e., subsynaptic nuclei) and are transcriptionally distinct from other myonuclei of the muscle fiber (Simon et al., 1992; Briguet and Ruegg, 2000). The implications from the increased number of nuclei is not yet clear, but we have previously shown a marked increase in AChR transcripts in *mdx* muscle (Pratt et al., 2015) and the increased nuclei could represent some sort of compensatory response. Maintaining a high concentration of AChRs at NMJ requires replacement of AChRs (Fambrough, 1979) by local insertion of AChRs into the postsynaptic membrane (Role et al., 1985). Furthermore, the mRNAs encoding the different subunits of the AChR are highly concentrated at synaptic sites in adult myofibers (Merlie and Sanes, 1985; Fontaine and Changeux, 1989; Brenner et al., 1990). Although the pathway remains unclear, evidence strongly suggests that transcriptional control of AChR genes in subsynaptic nuclei is crucial for proper clustering of AChRs (Ferraro et al., 2012). Muscle-specific kinase (MuSK) appears to be necessary for synapse-specific transcription (Herbst and Burden, 2000; Lacazette et al., 2003) and we have shown previously that MuSK is reduced in *mdx* muscle (Pratt et al., 2013).

Our study has several limitations. With our methods, we cannot distinguish myonuclei from satellite cells. We also cannot be positive that the nuclei are only myonuclei, as there are also nuclei from non-muscle cells associated with the NMJ. For example, non-mylenating Schwann cells, or terminal Schwann cells (TSCs), are located at the terminal motor nerve and their processes cover the terminal bouton. There is growing evidence that TSCs play an important role in synaptic transmission, synaptogenesis, and nerve regeneration (Balice-Gordon, 1996; Lubischer and Bebinger, 1999; Grady et al., 2005; Smith et al., 2013; Kang et al., 2014), particularly at the NMJ in *mdx* muscles (Pereira et al., 2001; Personius and Sawyer, 2005; Marques et al., 2006, 2007). By using a confocal Z-stacked image with boundaries limited to BTX staining, only nuclei within the sarcolemmal region should have been used in our analysis, but we cannot rule out the presence of TSCs without specific labeling for them.

Another limitation is that we did not examine *mdx* mice younger than 3 months of age. A “critical period” has been described for the *mdx* mouse, during which there is a peak in muscle weakness, myofiber necrosis and regeneration between the 2nd and 5th weeks of life (Dangain and Vrbova, 1984; Glesby et al., 1988; Muntoni et al., 1993; De la Porte et al., 1999; Chamberlain et al., 2007). Although *mdx* mice continues to show marked muscle weakness and susceptibility to contraction-induced injury throughout their lifespan, the critical period is a time that most closely mimics the histopathology seen in DMD.

In summary, the NMJ does not have a fixed, permanent structure, but instead shows plasticity in response to muscle injury, disease, and aging. We report here that, despite the tight coupling between presynaptic and postsynaptic structure reported for aging NMJs, this overlap between presynaptic and postsynaptic morphology (NMJ occupancy) is altered in the mouse model of Duchenne muscular dystrophy. This remodeling reflects plasticity in both pre- and postsynaptic contributors to NMJ structure, which could influence neuromuscular transmission and ultimately muscle function, however further study is needed to fully understand these structure-function relationships.

### Conflict of interest statement

The authors declare that the research was conducted in the absence of any commercial or financial relationships that could be construed as a potential conflict of interest.
